# Galectin 7 leads to a relative reduction in CD4+ T cells, mediated by PD-1

**DOI:** 10.1038/s41598-024-57162-3

**Published:** 2024-03-19

**Authors:** Guojin Wu, Wei Deng, Hsin-Yi Chen, Hye-Jeong Cho, Jaehyup Kim

**Affiliations:** 1 Department of Pathology, University of Texas Southwestern Medical Center, 5323 Harry Hines Blvd., Dallas, TX 75390-9072 USA; 2 Department of General Surgery, Beijing Friendship Hospital, Capital Medical University, Beijing, China

**Keywords:** Immunology, Tumour immunology

## Abstract

The role of glycan-binding proteins as an activator of immune regulatory receptors has gained attention recently. We report that galectin 7 reduced CD4+ T cell percentage in both in vitro culture and mouse tumor models. Immunohistochemical staining of esophageal cancer patient samples showed a lower percentage of CD4+ cells in the galectin 7 high area. The lack of CD4+ T cell depletion by galectin 7 in PD-1 knockout mice supports the role of PD-1 in mediating the effects of galectin 7. The binding assays demonstrate that galectin 7 binds to the *N*-glycosylation of PD-1 on N74 and N116 sites and leads to the recruitment of SHP-2. NFAT suppressive activity of galectin 7 was abrogated upon overexpression of the dominant negative SHP-2 mutant or inhibition of PD-1 by siRNA. Glycosylation of PD-1 has been reported to play a critical role in surface expression, stability, and interaction with its ligand PD-L1. This report further expands the significance of PD-1 glycosylation and suggests that galectin 7, a glycan-binding protein, interacts with the immune regulatory receptor PD-1 through glycosylation recognition.

## Introduction

Glycan is increasingly recognized as a major activation target for immune checkpoint receptors. The glycosylation of TIM3 is targeted by galectin 9, leading to TCR signal down-regulation^1^. Galectin 3 binds to LAG-3 by glycosylation recognition^2^. We further expand the list of interactions between immunomodulatory receptors and glycan binding lectins by discovering the interaction of galectin 7 with PD-1. PD-1 is a major immune checkpoint inhibitor, and monoclonal antibodies that prevent the PD-1 activation induce polyclonal expansion of peripheral T cells^3^, hyperactivation of naïve T cells^4,5^, and functional recovery of memory T cells that recognize cancer^6,7^. Clinical trials of PD-1 blockade have demonstrated improved survival and durable response in several solid organ tumors, including melanoma, lung cancer, renal cell carcinoma, and head and neck cancer^8–11^. Thus, identifying a novel PD-1 activation mechanism could enhance the outcomes of immune checkpoint receptor-targeted therapy.

In this study, we demonstrate that galectin 7 skews the distribution of T cells toward fewer CD4+ T cells. The syngeneic mouse tumor models confirmed that galectin 7 reduces the percentage of CD4+ T cells in WT mice but not in PD-1 KO mice, suggesting a potential role of galectin 7 in PD-1. Immunohistochemical staining of esophageal cancer patient samples also showed a negative correlation between galectin 7 expression and the percentage of CD4+ T cells. A co-immunoprecipitation study showed that galectin 7 interacts with PD-1 through *N*-glycosylation recognition, and NFAT reporter cell assay confirmed that PD-1 and SHP-2 are required for the NFAT suppressive effect of galectin 7.

## Results

### CD4+ and CD8+ T cells differ in their response to Galectin 7

**Figure 1:**
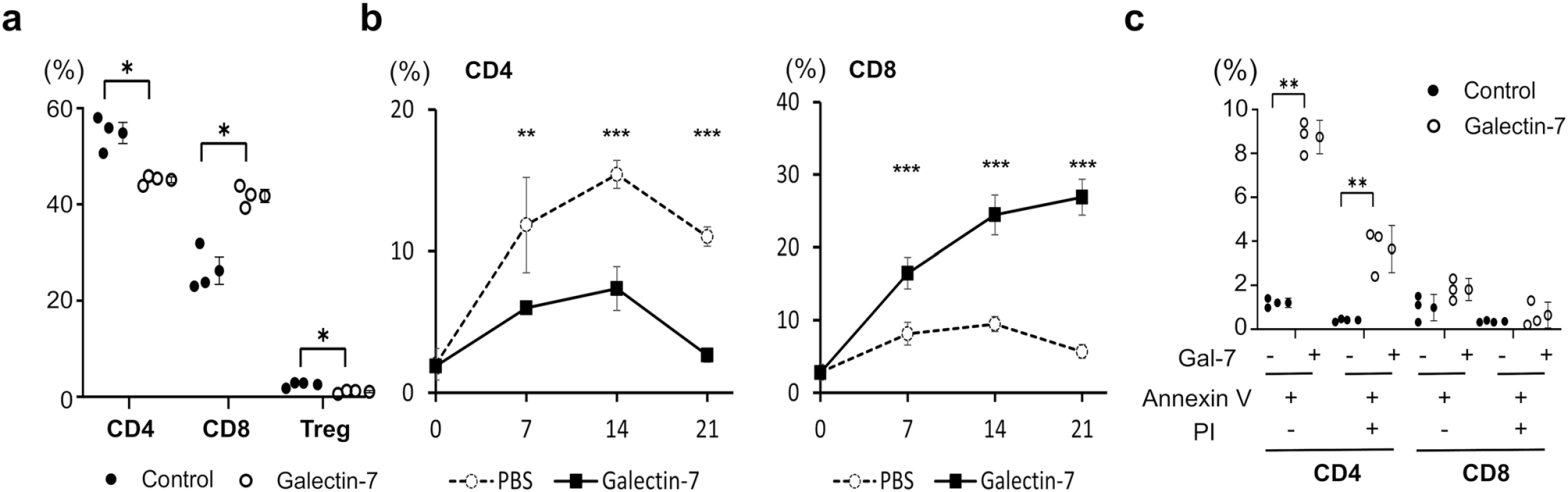
Galectin 7 treatment leads to a relative reduction in the percentage of CD4+ T cells. (**a**) Human PBMC were cultured in galectin 7 or BSA control for 7 days. The percentage of CD4+ , CD8+ T cells and regulatory T cells (CD4+ FoxP3 +) was quantified by flow cytometry (n = 3). (**b**) NSG mice were transplanted with human PBMC. From day 14 of the PBMC injection, the mice were treated with PBS or Galectin-7 (I.V. injection, 1.5 mg/kg) every 4 days. The human T cells in the peripheral blood (PB, depleted red blood cells) were detected by FACS with anti-hCD4 and anti-hCD8. (n = 4). (**c**) Activated human primary CD4+ and CD8+ T cells were treated with coated galectin 7 (20 μg/ml) or BSA control. Cells were stained with Annexin V FITC and propidium iodide to quantify apoptosis using flow cytometry. (n = 3). Error bars show mean ± SD.

Human PBMCs were cultured in the presence of human galectin 7 or BSA control for 7 days, and the relative percentages of CD4+ and CD8+ T cells among total lymphocytes were determined by flow cytometry. The result showed a relative decrease in the percentage of CD4+ T cells and a relative increase in the percentage of CD8+ T cells (Fig. 1a). To confirm the effect of galectin 7 on CD4+ and CD8+ T cells, we injected human PBMC into NSG mice. From day 14 of PBMC injection, mice received galectin 7 or PBS every 4 days. The results show that galectin 7 reduced the percentage of CD4+ T cells compared to the PBS injection group but increased the percentage of CD8+ T cells (Fig. 1b). To determine the mechanism, we performed the apoptosis assay using activated CD4+ and CD8+ T cells isolated from human PBMCs using magnetic bead-based sorting. The results showed a higher percentage of apoptosis of CD4+ T cells compared to CD8+ T cells in response to galectin 7 treatment (Fig. 1c).

### Galectin 7 reduces CD4+ T cells, which is abolished in the absence of PD-1

**Figure 2:**
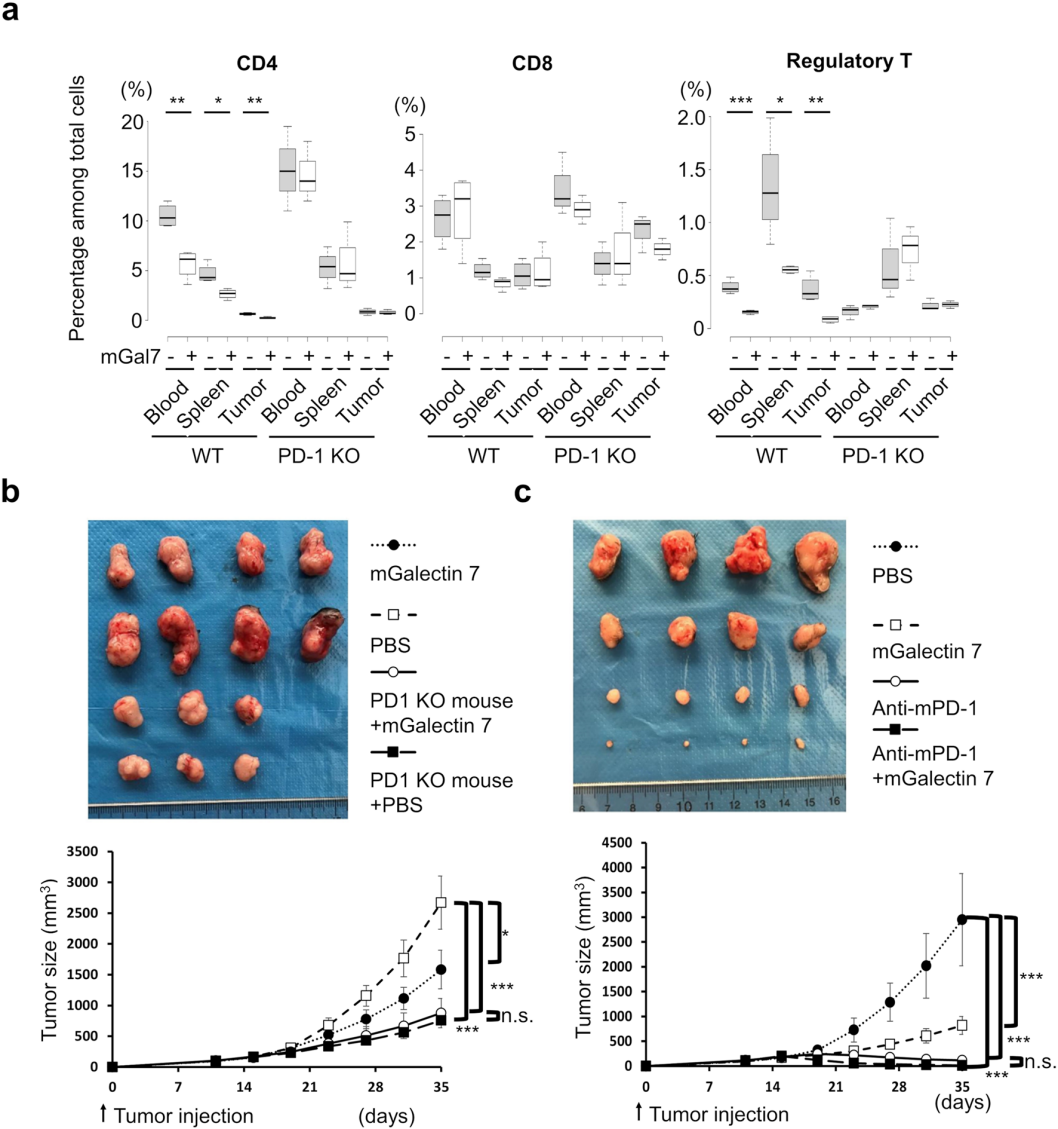
Galectin 7 reduced the percentage of CD4+ T cells and suppressed tumor growth. (**a**) WT C57BL/6 or PD-1 KO mice were injected with mouse colon cancer cell line MC38 at a dose of 1 × 10^5^. The mice received intravenous injections of mouse Galectin-7 (1.5 mg/kg) or PBS every 4 days from day 15 of tumor transplantation. On day 35, mice were sacrificed, and the percentages of CD4+ , CD8+ , and regulatory T cells were analyzed by flow cytometry. RBC depleted peripheral blood, spleen, and tumor cells were stained with anti-CD4, anti-CD8 and anti-CD25 antibodies for flow cytometry analysis. (CD4 group, t-test, *p* = 0.002, *p* = 0.017, *p* = 0.002 for WT), (Regulatory T cell group, t-test, *p* = 0.004, *p* = 0.049, *p* = 0.017 for WT), (n = 4 for WT, n = 3 for PD-1 KO). (**b**) Growth of MC38 cancer cells injected into WT or PD-1 KO mice. (one way ANOVA with Tukey HSD, *p* = 0.0289 for PBS vs. mGalectin 7, *p* = 5.96 × 10^−5^ for PBS vs. PD-1 KO, *p* = 1.03 × 104 for PBS vs. PD-1 KO with mGalectin 7, and *p* = 0.9657 for PD-1 KO vs. PD-1 KO with mGalectin 7 injection group), (n = 4 for WT, n = 3 for PD-1 KO). (**c**) MC38 cancer cells were injected into WT mice with an isotype control or anti-mPD-1 antibody. (one way ANOVA with Tukey HSD, *p* = 0.00018 for PBS vs. mGalectin 7, *p* = 1.1 × 10^−5^ for PBS vs. anti-mPD-1, *p* = 7.6 × 10^−6^ for PBS vs. mGalectin 7 and anti-mPD-1, *p* = 0.9889 for mGal7 vs. mGalectin 7 and anti-mPD-1), (n = 4). Error bars show mean ± SD.

We then tested the significance of galectin 7 activity on T cells in vivo using a syngeneic tumor model. The colon cancer cell line MC38 was injected into the flank area of WT and PD-1 knockout mice at a dose of 1 × 10^5^ cells. Fifteen days after tumor cell injection, the mice were treated with vehicle (PBS) or galectin 7 (1.5 mg/kg) every 4 days. At the endpoint (day 35), peripheral blood, spleen and tumors were harvested and subjected to flow cytometry analysis. In all examined tissues, mice treated with galectin 7 showed lower percentages of CD4+ T cells (Fig. 2a). CD4+ CD25 + regulatory T cells showed a similar trend to CD4+ T cells (Fig. 2a). Notably, galectin 7 treatment did not lead to reduced percentages of CD4+ T cells or regulatory T cells in PD-1 KO mice (Fig. 2a). CD8+ T cells did not exhibit differences between vehicle and galectin 7-treated groups for WT and PD-1 KO mice. Galectin 7 injection suppressed MC38 tumor growth in WT mice but not in PD-1 KO mice (Fig. 2b) or anti-mPD-1 antibody injected mice (Fig. 2c).

Syngeneic mouse tumor model using another mouse cancer cell line LL/2 engineered to overexpress mouse galectin 7 also showed a reduction in the percentage of CD4+ and CD4+ CD25 + regulatory T cells in the galectin 7 overexpression group, further supporting CD4+ T cell suppressive effect of galectin 7 (Supplemental Fig. 1a). Overexpression of galectin 7 suppressed tumor growth in the anti-mPD-1 antibody co-injection group (Supplemental Fig. 1b). The tumor suppressive effect of galectin 7 is dependent on the function of the immune system, as the overexpression of mouse galectin 7 in the LL/2 cell line enhanced tumor growth in NSG mice, unlike WT mice (Supplemental Fig. 1c).

### Galectin 7 expression level is correlated with tumoral T cells

**Figure 3:**
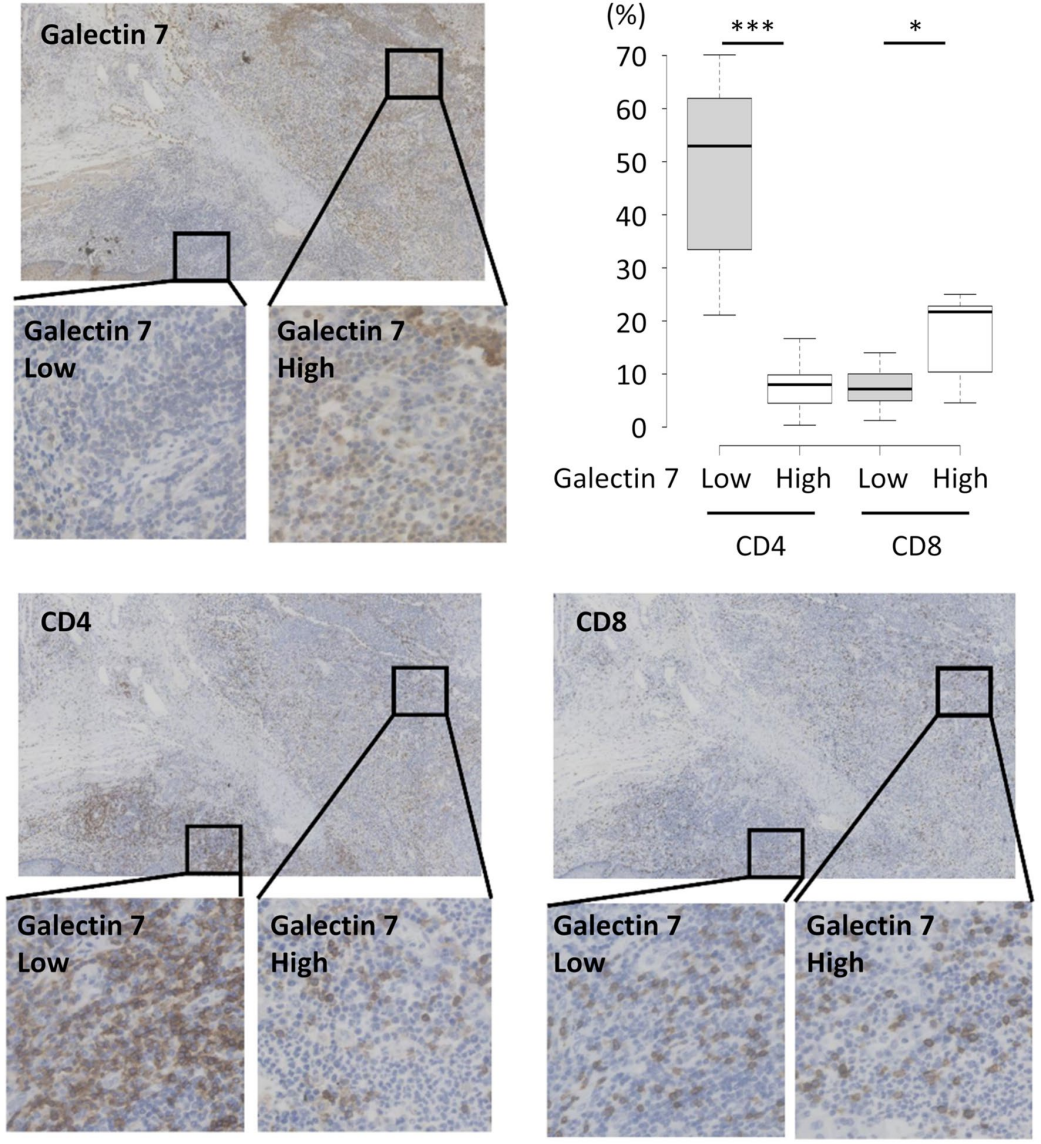
Galectin-7 shows an inverse correlation with intratumoral CD4 cells in human esophageal cancer tissue. Representative Immunostaining images show galectin 7, CD4, and CD8 staining. Galectin 7 high and low areas have been selected for visualization. The number of CD4+ and CD8+ cells within areas with high or low galectin 7 expression have been counted from twelve image sets, and the percentages are represented in the graph. *, *p* < 0.05; **, *p* < 0.01; ***, *p* < 0.001. Error bars show mean ± SEM.

To elucidate the significance of galectin 7 on tumoral T cells, immunohistochemical staining was performed on twelve tissue samples from esophageal cancer patients. Staining with anti-galectin 7, anti-CD4, and anti-CD8 antibodies showed that areas with low expression of galectin 7 had a higher percentage of CD4+ T cells and a lower percentage of CD8+ T cells (Fig. 3). Overall, these correlations are consistent with the results from mouse tumor models which showed reduction in CD4+ T cells upon galectin 7 injection or overexpression (Fig. 2 and Supplemental Fig. 1).

### Galectin 7 binds to PD-1 via recognition of N74- and N116-linked glycosylation

**Figure 4:**
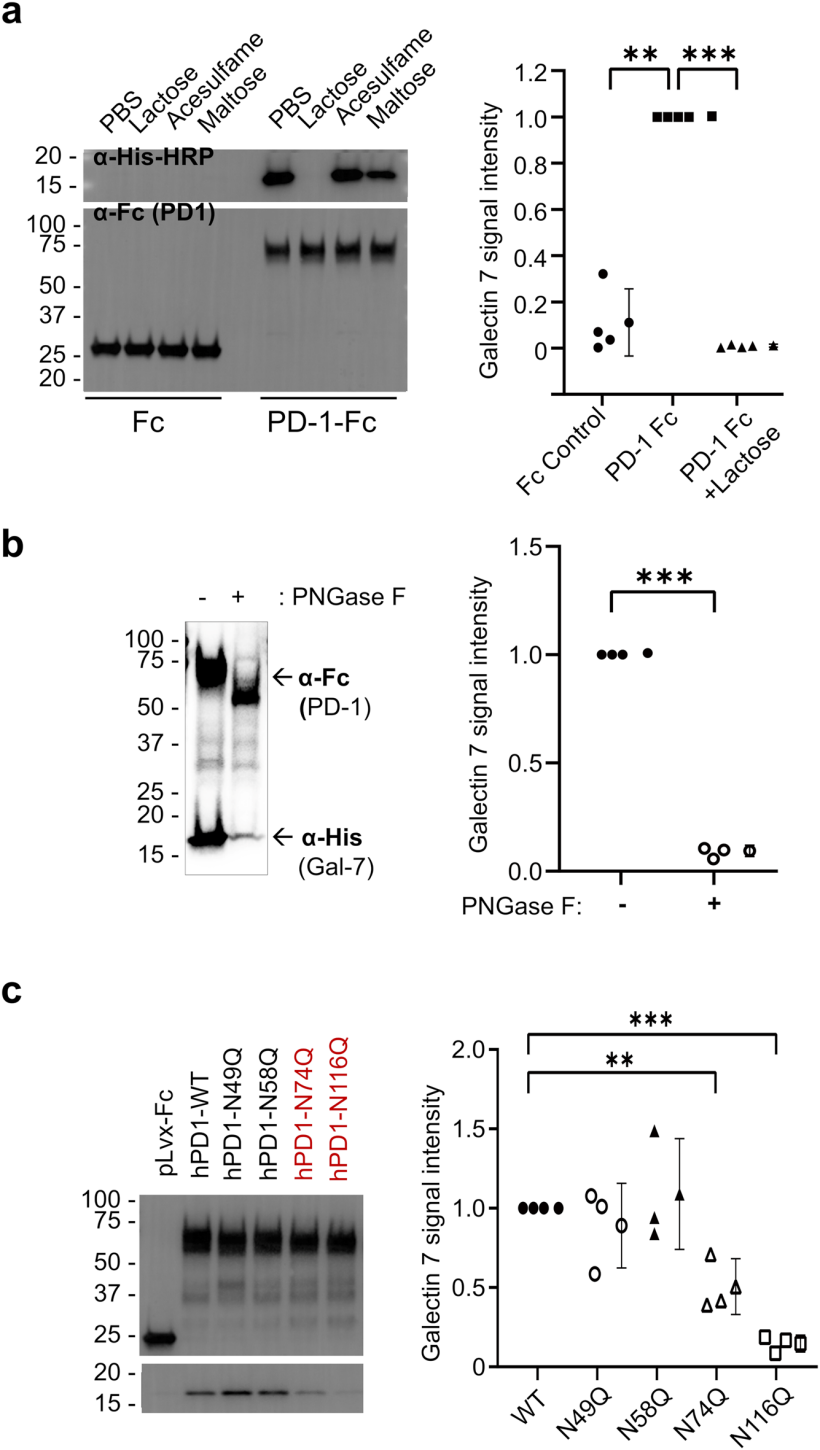
Galectin 7 binds to PD-1 by recognizing *N*-glycosylation on residues N74 and N116 of PD-1. (**a**) Cell lysates from 293 T cells overexpressing human-Fc-tagged PD-1 were incubated with His-tagged human galectin 7 (1 μM) and sugars (50 mM). After pull-down using protein A beads, purified proteins were subjected to Western blot with anti-human-Fc HRP (for PD-1) or anti-His-HRP antibodies (for galectin 7). The anti-His-HRP signal (galectin 7) was normalized against the anti-Fc-HRP signal (PD-1). (t-test, *** *p* = 6 × 10^−8^), (n = 4) (**b**) PD-1-Fc overexpressed 293 T cell lysates were treated with or without peptide-N-glycosidase F for 16 h. Subsequently, the lysate was incubated with recombinant His-tagged human galectin 7 (1 μM) for 2 h. After the pull-down, purified proteins were subjected to Western blot. The signal intensity of the PNGase F treatment group was normalized against the control. (t-test, *** *p* = 2 × 10^−4^), (n = 3) (**c**) Cell lysate from 293 T cells overexpressing human-Fc-tagged PD-1 wild type and PD-1 glycosylation site mutants were incubated with His-tagged human galectin 7 (1 uM). After pull-down using protein A beads, purified proteins were subjected to Western blot. (t-test, * *p* = 0.039, *** *p* = 6 × 10^−4^), (n = 3). Error bars show mean ± SD.

Based on the known biological role of galectin 7 as a carbohydrate-binding lectin, we sought to determine if galectin 7 binds to PD-1 by glycan recognition. A surface binding assay using 293 T cells transfected with PD-1 showed increased galectin 7 binding, suggesting PD-1 as the binding partner of galectin 7 (Supplemental Fig. 2). Lactose blocks the lectin activity of galectin 7, with its beta-galactoside structure serving as a natural ligand to the carbohydrate-binding domain of galectins. The addition of lactose abolished galectin 7 binding to PD-1 to a greater degree than maltose, a non-beta-galactoside containing sugar, or sugar analog Acesulfame K (Fig. 4a). This result indicates that the sugar-binding capacity of galectin 7 is necessary for the interaction with PD-1.

Next, we sought to determine if *N*-glycosylation mediates the binding since PD-1 is primarily N-glycosylated^12^. Removing *N*-glycosylation using PNGase F treatment significantly reduced galectin 7 binding to PD-1 (Fig. 4b), suggesting that N-linked glycans mediate the binding. To locate the *N*-glycosylation site involved in galectin 7 binding, we created PD-1 with a mutation in one of the known *N*-glycosylation sites, which play critical roles in PD-1 stability and PD-L1 binding^12,13^. Mutation of PD-1 at N74 or N116 considerably reduced the binding of galectin 7, identifying *N*-glycosylation of these two sites as the target of galectin 7 (Fig. 4c).

### Galectin 7 promotes SHP2 recruitment by PD-1 and dampens TCR signaling

**Figure 5:**
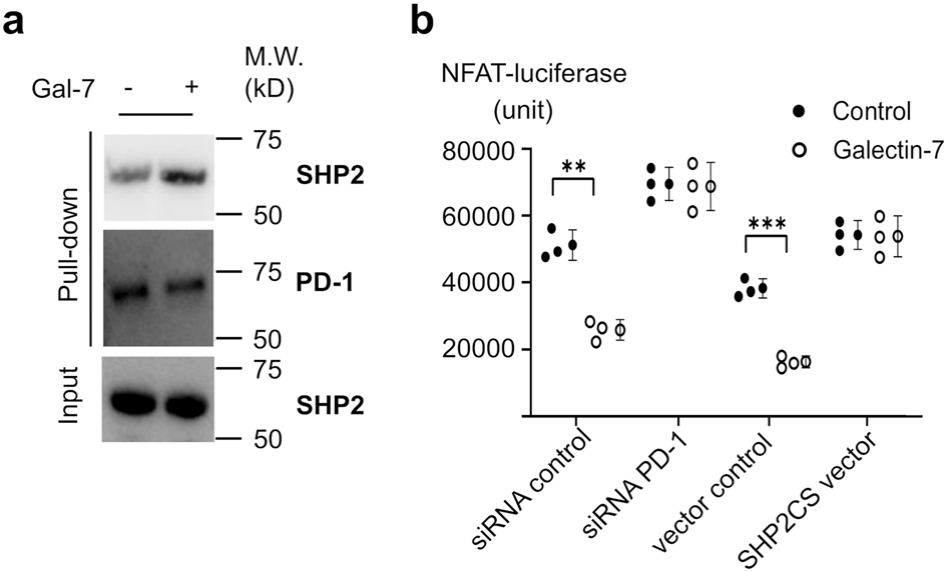
Galectin 7 binding to PD-1 leads to SHP-2 recruitment and suppression of NFAT. (**a**) Jurkat cells transduced with lentivirus encoding Fc tagged human PD1 were treated with BSA or Galectin-7 (1 μM), and the interaction of PD1 and shp2 was analyzed with co-IP. (**b**) Jurkat NFAT reporter cells were transfected with control siRNA or siRNA against PD1. Another set of Jurkat NFAT reporter cells was transduced to overexpress WT or dominant negative shp2 mutant (SHP2CS). Cells were cultured in a plate coated with anti-CD3 in the presence of BSA or Galectin-7 (1 μM). The activation of NFAT signaling was analyzed by quantifying the luciferase activity using a plate reader. (t-test, ** *p* = 0.0023, *** *p* = 0.00078), (n = 3). Error bars show mean ± SD.

SHP-2 is a key downstream effector of PD-1, which gets recruited to PD-1 cytoplasmic tails upon ligand engagement. SHP-2 suppresses the TCR pathway upon recruitment to PD-1, promoting an exhausted T cell phenotype^14–16^. We tested if galectin 7 treatment affected the recruitment of SHP-2 to PD-1. Using a lentiviral transduction method based on an established protocol^17^, hFc-PD-1 protein was overexpressed in Jurkat cells. Jurkat cells were activated and cultured in the presence or absence of galectin 7 and then lysed for hFc-PD-1 pull down by protein A. Galectin 7 treatment enhanced SHP-2/PD-1 co-immunoprecipitation (Fig. 5a), suggesting that galectin 7 led to PD-1 activation.

To further probe the effect of galectin 7 on modulating T cell response via PD-1, we used Jurkat reporter cells stably expressing luciferase under the control of the Nuclear Factor of Activated T cells (NFAT). NFAT is a family of transcription factors that cooperate with other proteins to regulate T cell fate and functions upon TCR stimulation. Jurkat cells upregulate PD-1 upon activation, which can provide a helpful model to study PD-1^18^. Galectin 7 suppressed the NFAT transcription activity, and the inhibition of PD-1 using siRNA or blockade of SHP-2 activity using a dominant-negative SHP-2 mutant (SHP2CS) blunted the effect of galectin 7 (Fig. 5b). Altogether, these results support a model that galectin 7 binds to PD-1, leading to its activation and suppressing TCR signaling.

## Discussion

This study demonstrates that galectin 7 binds to PD-1 through glycosylation recognition, leading to SHP-2-mediated TCR suppression. Previous studies have established a critical role of PD-1 Glycosylation in the expression and stability of PD-1^12^. Additionally, the recognition of PD-1 by PD-L1 is highly dependent on PD-1 *N*-glycosylation, especially at N58 location^12,13^. We discovered that galectin 7 binding to PD-1 depends on glycosylation of N74 and N116 locations, which is distinct from the PD-L1 binding mechanism. This study expands the significance of PD-1 glycosylation as a binding target of glycan recognition proteins.

Galectins are a class of glycan recognition proteins characterized by a carbohydrate recognition domain (CRD) with varying affinity for β-galactose^19^. Recently, the role of galectins as a regulator of immune regulatory receptors has begun to garner attention. Galectin 9 as a ligand of immune regulatory receptor TIM-3 has been well established^1,20^. It was also reported that galectin 3 binds to another immune regulatory receptor, Lymphocyte-activation gene 3 (LAG3), leading to its activation^2^. Yang et al. identified the binding of galectin 9 with PD1 in addition to previously known TIM3^21^. However, direct activation of PD-1 by galectin 9 was not reported. Instead, the competition of PD-1 with TIM-3 for galectin 9 binding resulted in reduced TIM-3 lattice formation and activation. These findings suggest that the interaction between sugar-binding lectin and immune receptors might be more prevalent than recognized. Our discovery further expands the galectin-immune regulatory receptor interaction repertoire and accentuates galectins’ immune-regulating role.

Galectin 7 is highly expressed in the stratified epithelium, especially the skin^22–24^. Combined with its role as a PD-1 activator, galectin 7 has the potential to play a critical role in skin immune tolerance. Keratinocytes upregulate PD-L1 during inflammation, which is considered a primary mechanism of immune regulation in the skin via interaction with PD-1^25–27^. However, the incidence of cutaneous immune-related adverse events differs significantly between PD-L1 blockade therapy (up to 20%) and PD-1 blockade (34–42%)^28^, suggesting that PD-L1 may not be the sole binding partner of PD-1 in the skin. It would be interesting to study if Galectin 7 can fill the role of a complementary PD-1 activator in the skin.

In addition to its physiological function, galectin 7 may play a role in cancer. While galectin 7 expression is restricted to epithelial cells in normal tissues, some types of cancer ectopically overexpress galectin 7^29^. Analysis of the cancer gene expression database also shows a high level of galectin 7 in some types of carcinoma compared to other galectins (Supplemental Fig. 3)^30^. Moreover, cancer treatments can enhance galectin 7 expression^31^. Previous studies have shown that the effect of galectin 7 on cancer progression appears to be different between the histologic types^22^. A higher level of galectin 7 was associated with tumor suppression in neuroblastoma and colon cancer^32,33^ but enhanced the progression of breast cancer and lymphoma^31,34–36^. Recently, galectin 7 has been reported to convert the tumor immune environment of colorectal cancer with microsatellite instability and improve responsiveness to anti-PD-1 therapy^37^. This finding aligns with our observation and further supports the immune modulatory effect of galectin 7 in tumors.

In this study, the injection of galectin 7 reduced CD4+ T cells and suppressed tumor growth in the MC38 syngeneic mouse tumor model using WT mice but not in PD-1 KO mice. The observed reduction in the percentage of CD4+ FoxP3 + regulatory T cells, while sparing CD8+ T cells, can lead to de-suppression of anti-tumor immune response and reduce tumor growth. These results can help explain the discrepancy between different types of cancers. For example, in tumors with reduced infiltration of regulatory T cells, galectin 7 can be less effective in restoring anti-tumor immune response and even enhance tumor cell growth, as observed in the NSG mouse model (Supplemental Fig. 1c). Local changes in glycome composition or the presence of another galectin 7 binding protein can also affect the tumor response.

Immunohistochemical staining of samples from esophageal cancer patients showed that galectin 7 expression level was negatively correlated with the percentage of CD4+ T cells. In contrast, the percentage of CD8+ T cells was higher in areas with high galectin 7 expression levels (Fig. 3). Combined with results from syngeneic mouse tumor models (Fig. 2 and Supplemental Fig. 1), these findings suggest that galectin 7 can impact tumor immune response with potential clinical impact.

A difference in lectin binding between T cell subsets is not without precedence. Cabral et al. reported distinct surface glycosylation patterns associated with CD4+ Treg, with a subpopulation displaying high lectin binding and enrichment of surface receptors, including PD-1^38^. In the case of galectin 1, Th2 cells have higher expression of ST6Gal1, leading to increased alpha2-6 linked sialic acid on surface glycoproteins and resistance to galectin 1 mediated cell death^39^. Differential binding of galectin 7 to TCR receptor on CD4+ versus CD8+ T cells has been reported previously^40^. Galectin 7 showed more significant effects on the CD4+ T cells in our study, which aligns with previous reports about T cell subset dependent differences in glycosylation. If confirmed, the difference in PD-1 glycosylation between CD4+ and CD8+ T cells could lead to a novel therapeutic approach allowing T cell subset targeted PD-1 regulation.

Lectins are specific to glycan structures rather than protein structures, unlike traditional receptor-ligand pairs. Naturally, they can interact with multiple proteins that share similar glycan structures, and isolating the contribution of a single binding partner remains a significant challenge. For instance, galectin 7 has previously been reported to bind TCR^40^. Since PD-1 signaling suppresses the TCR pathway, the binding of galectin 7 to TCR can complicate the PD-1 signaling pathway analysis. We focused on the effect of PD-1 and SHP-2 in NFAT reporter cell assay as the signaling pathway further down could be affected by other galectin 7 binding proteins. The results confirm that PD-1 siRNA or dominant negative SHP2 mutant does not suppress NFAT signal in response to galectin 7, attesting to the contribution of the PD-1 pathway. Furthermore, the lack of galectin 7 mediated changes in tumor infiltrating T cells in the PD-1 KO syngenetic mouse tumor model also attests to the significant role of PD-1 in the lymphocyte suppressive function of galectin 7.

This study focused on PD-1 activation as a mechanism for the observed immunological effect of galectin 7. However, the effects of galectin 7 likely involve multiple players and are more nuanced than simple immunosuppression by PD-1 activation. We recently reported that galectin 7 is a ligand of LILRB3, an immunoregulatory receptor expressed on myeloid cells^41^. LILRB3 is absent in lymphocytes, so this interaction is unlikely to play a role in the T cell changes reported in this study. Still, alteration of myeloid cells via LILRB3 activation could affect tumor immunology. The glycosylation-specific nature of galectin 7 binding can potentially affect other immune regulatory receptors and warrants further study.

Collectively, our data demonstrate that galectin 7 binds to PD-1 and leads to the suppression of the TCR pathway while contributing to the reduction of CD4+ T cells. This effect can shape the immune landscape in galectin-7-rich environments such as the skin or ectopic expression by cancer cells.

## Methods

### Mice

C57BL/6J mice were purchased from and maintained at the animal core facility of the University of Texas (UT) Southwestern Medical Center. NOD.Cg-Prkdc^scid^H2-Ab1^tm1Doi^Il2rg^tm1Wjl^/SzJ (NSG) and PD-1 knockout mice (B6.Cg-Pdcd1tm1.1Shr/J) were purchased from Jackson Laboratory. Mouse colon cancer cell line MC38 (Kerafast Cat# ENH204-FP) was subcutaneously injected into the flank of WT or PD-1 knockout C57BL/6J mice between 8 and 10 weeks of age at a dose of 1 × 10^5^ per mouse. Every 4 days from day 15 after tumor transplantation, the mice were treated with control mouse IgG or anti-mouse PD-1 at a dose of 5 mg/kg by retro-orbital injection into mice anesthetized with isoflurane. The galectin 7 treatment group received mouse Galectin-7 (Abcam Cat# ab134633) at 1.5 mg/kg, while the control group received PBS only control.

### Plasmids

*PDCD1 was* cloned from human T cell cDNA. The dominant-negative Src homology region 2 domain-containing phosphatase-2 (*SHP2*) mutant (*SHP2CS*) was a gift from Chengcheng Zhang Lab (UT Southwestern Medical Center). *SHP2CS* was cloned into the pLVX-IRES-ZsGreen1 vector (Takara Bio Cat# 632187). *PDCD1* was fused with human Fc and cloned into the plasmid pFuse-hIgG1-Fc1 (InvivoGen). Mouse galectin-7 (*LGALS7*) cDNA (VectorBuilder) was cloned into a pLVX-puro vector (Takara Bio #632164). The PD-1 glycosylation site mutants (N49Q, N58Q, N74Q, N116Q) were made with Q5 site-directed mutagenesis kit (New England Biolabs Cat#E0554S), using the wild type constructs (pFuse-PD1-Fc or pLVX-PD1-Fc) as templates.

### T cell preparation

Primary human T cells were isolated from healthy donor-derived buffy coats. Briefly, peripheral blood mononuclear cells (PBMC) were separated by density gradient using Ficoll-Paque (Cytiva Lifesciences Cat# 1714-4003) and treated with ACK lysis buffer (KD Medical Cat# RGF3015) to remove RBCs. Cell counting was performed by TC20 automated cell counter (BioRad). The cell viability after isolation was greater than 95% in most cases. Cells were cultured in RPMI1640 media (Sigma), supplemented with 10% fetal bovine serum (Hyclone Cat#SH30910.03) and 1% non-essential amino acids (Hyclone Cat#SH30238.01).

For T cell activation, primary human CD4+ T cells or CD8+ T cells were co-cultured with anti-CD3/CD28 Dynabeads (Thermo Fisher Scientific Cat# 11161D) in the presence of 10 ng/ml IL-2 (Peprotech Cat# 20-002-250UG), and the activated Dynabeads were removed 2 days later. T-cells were expanded for an additional week in the presence of IL-2.

For T cell isolation from the tumor model, peripheral blood was harvested by cardiac puncture, and RBCs were removed by incubation with ACK lysis buffer. Spleen cells were harvested by dissecting the spleen, followed by mechanical dissociation using slides. Primary tumors were removed and mechanically dissociated. The tissue was washed with PBS and placed in a collagenase-dispase medium (Sigma-Aldrich Cat# 10269638001) at 37 °C for 90 min. Cells were passed through a 70-µm strainer for further flow cytometry analysis or sorting.

### Western blotting and co-immunoprecipitation

To determine if galectin 7 binds to PD-1 by glycan recognition, cell lysate from 293 T cells (ATCC Cat# CRL-3216) transfected with hFc-tagged PD-1 or empty Fc-tagged vector were incubated for 2 h with His-tagged Galectin-7 (1 uM) in buffers of PBS, lactose (50 mM), Acesulfame K (50 mM), and Maltose (50 mM), respectively. Subsequently, the lysate was incubated with protein A magnetic beads (New England Biolabs) for 12 h. Bound proteins and beads were washed 3 times with PBS-T buffer (PBS containing 0.1% Tween 20), eluted in 4 × SDS sample buffer for western blotting, and separated by SDS-PAGE. After transfer to PVDF membranes, the protein was detected with specific primary antibodies and HRP-conjugated secondary antibodies. The image was acquired using ChemiDoc MP and Image Lab software (BioRad).

To confirm that PD-1 binds mediated by *N*-glycosylation, 293 T cells that overexpress hFc tagged PD-1 were lysed in IP buffer, and lysate was incubated for 16 h with or without PNGase F (New England Biolabs) at 37°. To detect the binding of PD-1 and Galectin-7, the lysate was incubated with His-tagged-Galectin-7 recombinant protein (1 uM) for 2 h at 4°. Subsequently, it was followed by co-immunoprecipitation and western blotting.

To confirm the binding of PD-1 mutants and Galectin-7 protein, Jurkat cells (ATCC Cat# TIB-152) were stimulated with CD3 antibodies (BioLegend Cat# 317302) at 10 ug/ml concentration for 2 days. Jurkat cell lines that expressed hFc-tagged PD-1 wild type or mutant protein were lysed in IP buffer (FisherScientific Cat#PI87788), and lysate was incubated for 2 h with His-tagged-Galectin-7 recombinant protein (SinoBiological Cat#12000-H07E-100) (1 uM) at 4°. Subsequently, it was followed by co-immunoprecipitation and western blotting.

The primary antibodies used were anti-h/m Galectin-7 (Novus Cat# NB100-380; 1:1000), anti-actin (BioLegend Cat# 664802; 1:10000), and anti-SHP-2 (Cell Signaling Technology Cat# 3397 T; 1:1000). Secondary antibodies used include mouse anti-goat IgG-HRP (Santa Cruz Biotechnology Cat# sc-2354), goat anti-rabbit IgG-HRP (Abcam Cat# ab6721), goat anti-rat IgG-HRP (BioLegend 405405), and rabbit anti-mouse IgG-HRP (Abcam Cat# ab6728).

### Virus production and infection

Lentiviruses were produced with viral constructs (pLVX-Puro plasmids), psPAX2 and pMD2G by co-transfection into Lenti-293 T cells using polyethyleneimine (PEI). The virus supernatant was collected 72 h after transfection and precipitated using PEG-it (System Biosciences Cat#LV810A1). Cells were centrifugated with virus supernatant in the presence of polybrene (Millipore Sigma Cat#TR1003G, 8 ug/ml) at 800 g for 30 min and incubated for 3 days. For selection, cells were cultured in complete media containing puromycin.

### NFAT signaling analysis

Jurkat-Lucia NFAT reporter cells (Invivogen Cat#jktl-nfat) were transfected with *PDCD1* specific esiRNA (Millipore Sigma Cat# EHU146521) or control esiRNA (Millipore Sigma Cat# EHUEGFP) with GenMute™ siRNA Transfection Reagent for Jurkat Cells (Signagen Cat# SL100568-JURKAT). Two days after transfection, NFAT signaling was activated with coated anti-CD3 (BioLegend Cat# 317302) in the presence or absence of Galectin 7 and luciferase activity in the supernatant was analyzed by adding substrate (InvivoGen) according to the manufacturer’s protocol and quantifying luminescence signal by plate reader.

To introduce *SHP2CS*, Jurkat-Lucia NFAT cells were infected with lentivirus expressing *SHP2CS*. Reporter cells were cultured for 2 weeks before being activated with coated anti-CD3 to stimulate NFAT signaling.

### Flow cytometry

Primary antibodies used for this study includes Annexin V-FITC (Biolegend Cat# 640906), anti-PD-1-PE (BioLegend Cat# 621607), anti-human CD8-PE (BioLegend Cat# 344706), anti-mouse CD4-PE (BioLegend Cat# 100408), anti-mouse CD25-PE (BioLegend Cat# 102012), anti-mouse FOXP3-Alexa Fluor® 488 (BioLegend Cat# 126405), anti-mouse CD8-APC (BioLegend Cat# 100711), anti-mouse PD-1-PE (BioLegend Cat# 135205), IgG isotype control-PE (BioLegend Cat# 400907), anti-human CD4-APC (eBioscience Cat# RPA-T4). FoxP3 staining used FoxP3 buffer (BioLegend Cat#421403). Samples were analyzed using FACSCalibur and FlowJo software.

### Immunohistochemical (IHC) staining

Tumor tissue from esophageal cancer patients was fixed with 4% neutral buffered formalin for 24 h, followed by paraffin wax embedding. Serial paraffin sections cut with 5-μm thickness were stained for CD4, CD8 and Galectin-7. Briefly, tumor tissue sections were incubated with proteinase K solution for 5 min, and endogenous peroxidase was blocked with 3% hydrogen peroxide. The slides were incubated with antibodies against CD4 (ab133616, 1:200, Abcam), CD8 (ab101500, 1:200, Abcam) or Galectin-7 (K107200P, 1:100, Solarbio) overnight. HRP-anti-Rabbit (SE134, Solarbio) was applied after primary antibody incubation. Tissue staining was visualized with a DAB substrate chromogen solution, followed by hematoxylin counterstaining. The percentages of CD4 and CD8 positive cells were counted manually within the image.

### Statistics

Prism 9.0 (GraphPad Prism) was utilized for statistical analysis and visualization. BoxPlotR was also used for visualization. For pairwise comparisons, a t-test was used to determine the *p*-value. Tumor sizes in the mouse tumor models were analyzed by one-way ANOVA and post-hoc Tukey’s honestly significant difference (HSD) test for multiple comparisons. *P* was set at 0.05. *P* values less than 0.01 or 0.001 were marked with double or triple asterisks for visualization.

### Study approval

All animal experiments in this study were approved by the UT Southwestern Institutional Animal Care and Use Committee (animal protocol number 2020-102891) and were conducted in accordance with ARRIVE guidelines. Histological analysis of cancer patient samples was performed using de-identified leftover surgical samples following surgery at Beijing Friendship Hospital, Capital Medical University. All patients and donors signed the informed consent forms. The related research was approved by the Clinical Research Ethics Committee of Capital Medical University. All methods were performed in accordance with the relevant guidelines and regulations.

### Supplementary Information

Supplementary Information 1.

## Data Availability

The data generated in this study are available upon request from the corresponding author.
