# Supplementary material for: Galectin 7 leads to a relative reduction in CD4+ T cells, mediated by PD-1
Source: Sci Rep. 2024 Mar 19;14:6625. doi: 10.1038/s41598-024-57162-3 (PMC10951237; doi:10.1038/s41598-024-57162-3)
Supplement: Supplementary file 1 — Supplementary Information 1. [file 41598_2024_57162_MOESM1_ESM.pdf]

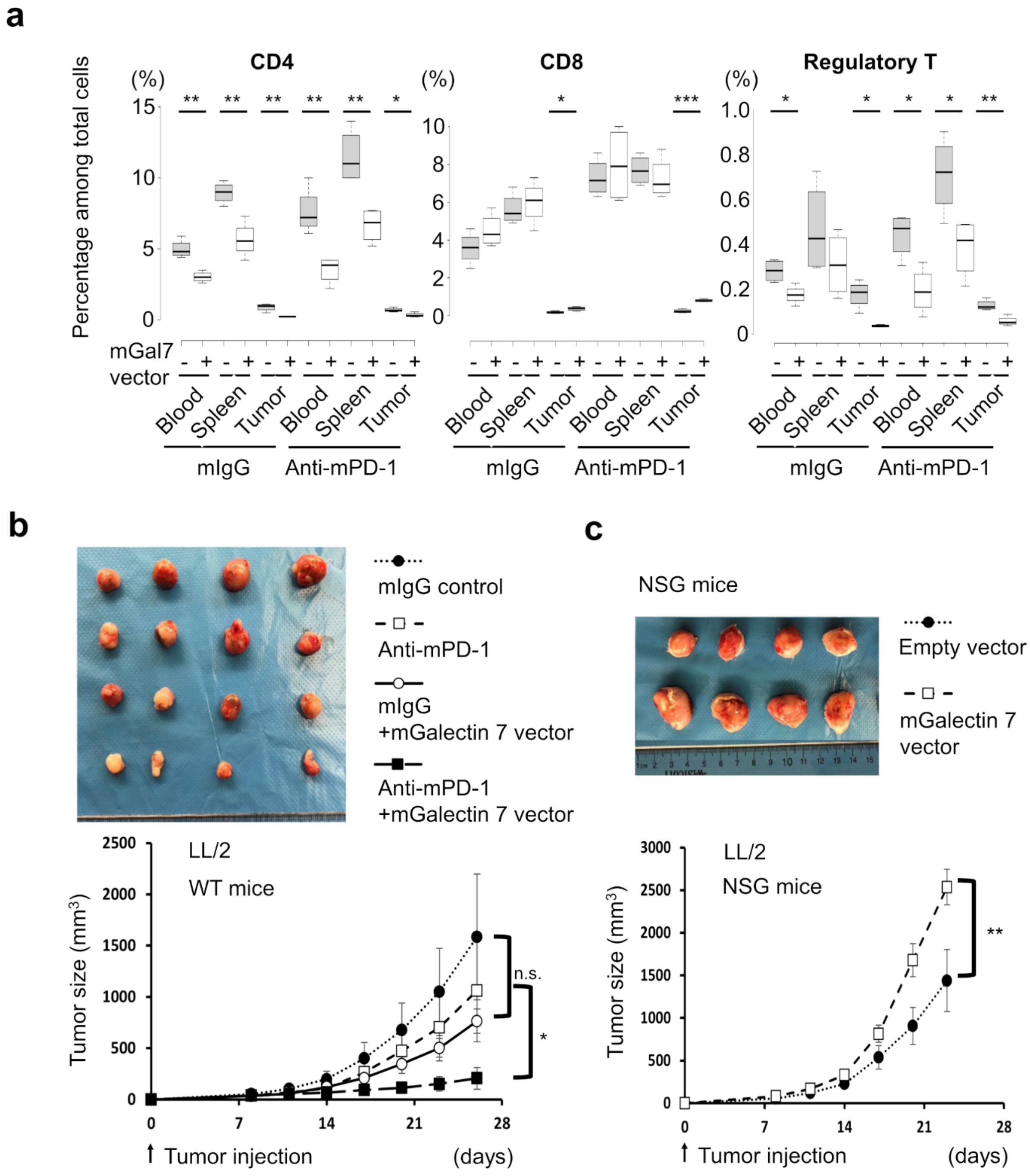

Supplemental Figure 1. Galectin 7 reduces CD4+ T cells in the LL/2 syngeneic mouse model and requires a functional immune system for tumor suppression. LL/2 cell line was transduced with lentivirus overexpressing mouse galectin 7 or empty vector control. 1x10^6 cells were injected into the flank area of C57BL/6J mice (8–10 weeks old). One week later, the

C57BL/6J mice were treated with control mouse IgG or anti-mouse PD-1 (InvivoGen) at a dose of 5 mg/kg by retro-orbital injection. On day 26, mice were sacrificed, and the percentage of CD4+, CD8+, and regulatory T cells were analyzed by flow cytometry. RBC-depleted peripheral blood, spleen, and tumor cells were stained with anti-CD4, anti-CD8 and anti-CD25 antibodies for flow cytometry analysis. (CD4 group, t-test, $p = 0.004$, 0.007, and 0.014 for control antibody group, $p=0.009$, 0.008 and 0.015 for anti-mPD-1 injection group), (Regulatory T cell group, t-test, $p = 0.018$, 0.259, and 0.020 for control antibody group, $p= 0.013$, 0.026, and 0.005 for anti-mPD-1 injection group), (n=4). **(b)** LL/2 cancer cells transduced with an empty vector or mGalectin 7 overexpression vector were injected into WT mice. (One way ANOVA with Tukey HSD, $p = 0.058$ for mIgG control vs. mIgG control with mGalectin 7 vector, $p = 0.04732$ for anti-mPD-1 with empty vector vs. anti-mPD-1 with mGalectin 7 vector), (n=4). **(c)** LL/2 cancer cells transduced with an empty vector or mGalectin 7 overexpression vector were injected into NSG mice. (t-test, $p = 0.004$), (n=4). Error bars show mean ± SD.

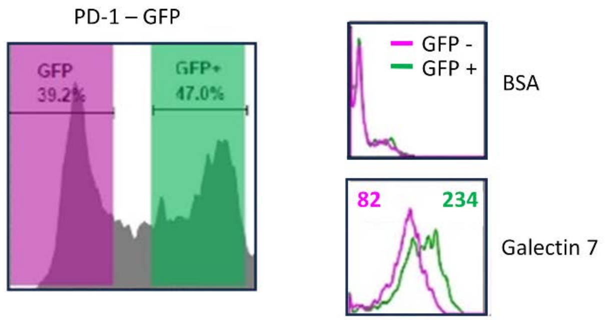

Supplemental Figure 2. Galectin 7 surface binding assay. 293T cells were transfected with a plasmid encoding pLVX-IRES-zsGreen or co-transfected with pLVX-IRES-zsGreen and CMV5.1-PD-1. Cells were incubated with galectin 7 or BSA at 1uM concentration in PBS for 1 hour at 4 degrees, then fixed by 10% formalin treatment for 30 minutes. After washing with PBS containing 0.2% BSA, cells were stained with anti-galectin 7 antibody conjugated with Alexa Fluor 647. Numbers denote the MFI for GFP+ (transfected cells) and GFP- (non-transfected cells) populations.

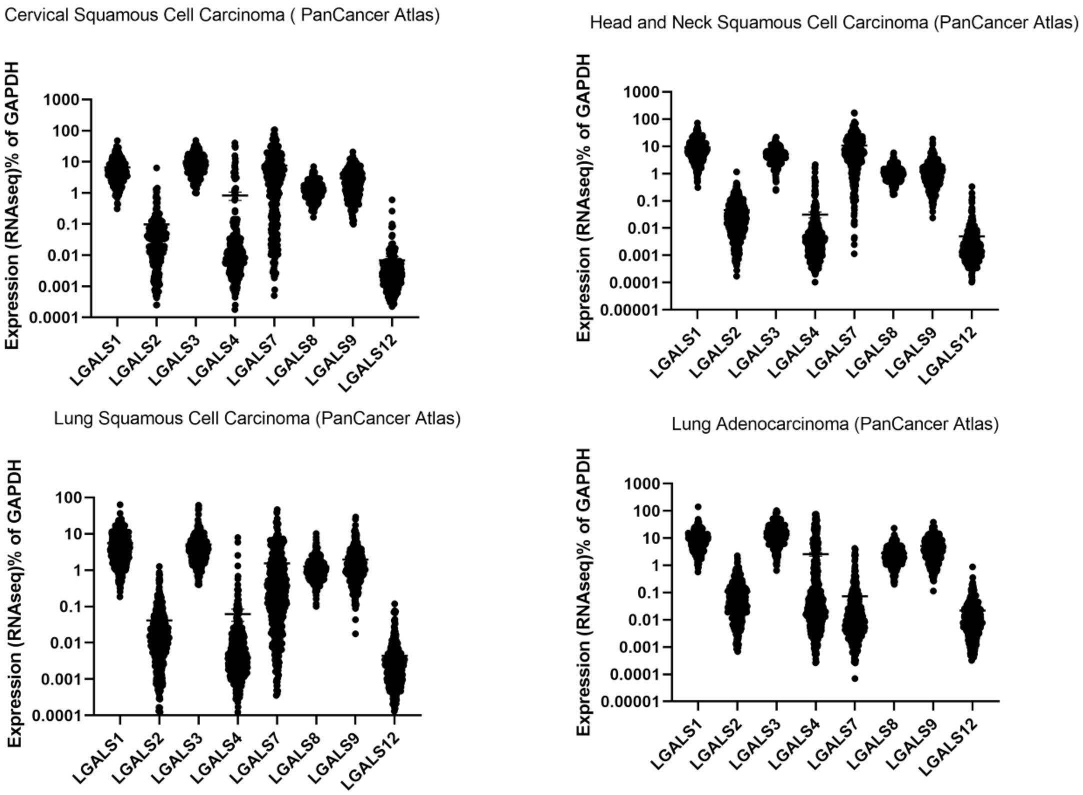

Supplemental Figure 3. Galectin expression level in selected cancer. RNA sequencing data from four cancer types (Cervical squamous cell carcinoma, Head and neck squamous cell carcinoma, Lung squamous cell carcinoma, and lung adenocarcinoma) were extracted from the cBioPortal public database. Galectin transcriptome level was normalized against GAPDH and shown as the percentage of the read of GAPDH.

Supplementary Methods

Mouse

For the LL/2 cell line model, 1×10^6 LL/2 cells (AddexBio Cat# C0016030/5000) transduced with lentivirus encoding mouse galectin 7 or empty vector control were subcutaneously injected into the flank of C57BL/6J mice (8–10 weeks old). One week later, the C57BL/6J mice were treated with control mouse IgG or anti-mouse PD-1 (InvivoGen Cat# MPD1MAB1510) at a dose of 5 mg/kg by retro-orbital injection into mice anesthetized with isoflurane. Mice were matched for gender and age in each experiment.

Western Blot

Recombinant His-tagged human galectin 7 (SinoBiological Cat#12000-H07E-100) and mouse galectin 7 (MyBioSource Cat#MBS204318) have been used as positive controls. HaCaT cells were obtained from AddexBio (Cat# T0020001). Cells were lysed in an IP buffer and subjected to Western blot. Proteins were stained with anti-h/m Galectin-7 (Novus Cat# NB100-380; 1:1000) or anti-actin (BioLegend Cat# 664801; 1:10000), followed by secondary antibody staining with goat anti-rabbit IgG-HRP (Abcam Cat# ab6721) or goat-anti-rat IgG-HRP (BioLegend Cat# 405405).

PCR

mRNA was isolated from cells using a Rneasy Mini kit (Qiagen, Cat#74104). cDNA was synthesized using High-Capacity Reverse Transcription Kit (Applied Biosystems, Cat#4368814). The following primers (Sigma) were used for PCR: 5`-GGGTCGACCTCGAGGCCACCATGTCTGCTACCCAGCACAAG-3’ and 5`-GGGGTACCGAAGATCTTCACTGAATGCAGCTG-3’ to detect mouse galectin-7, 5`-GAAGATCTGCCACCATGTCCAACGTCCCCCAC-3’ and 5`-GGGGTACCGAAGATCCTCACGGAGTCCAGC-3` to detect human galectin-7, 5`-GATTTGGTCGTATTGGGCGC-3` and 5`-TTCCCGTTCTCAGCCTTGAC-3` to detect human

GAPDH. The mRNA levels were normalized to the level of GAPDH present in the same sample. For calculating the copy number of target mRNAs, PCR products were purified by gel extraction and used as standard samples for normalization in qPCR analysis.

Flow cytometry

293T cells were transfected with plasmids encoding pLVX-IRES-ZsGreen only or co-transfected with pLVX-IRES-ZsGreen and CMV5.1-PD1. Cells were incubated with Galectin-7 (1uM) in PBS with 1% BSA for 1 hour at 4 degrees Celsius, then fixed with 10% formalin for 30 minutes. After washing with PBS with 0.2% BSA, the cells were stained with anti-Galectin-7 Alexa Fluor 647 (RnD Systems, Cat# 13392R).
